# Assessment of Immune Cell Activation in Pemphigus

**DOI:** 10.3390/cells11121912

**Published:** 2022-06-13

**Authors:** Anna Kowalska-Kępczyńska, Mateusz Mleczko, Weronika Domerecka, Dorota Krasowska, Helena Donica

**Affiliations:** 1 Department of Biochemical Diagnostics, Chair of Laboratory Diagnostics, Medical University of Lublin, 20-081 Lublin, Poland; helena.donica@umlub.pl; 2 Chair and Department of Dermatology, Venerology and Paediatric Dermatology, Medical University of Lublin, 20-081 Lublin, Poland; mateusz.p.mleczko@gmail.com (M.M.); dorota.krasowska@umlub.pl (D.K.); 3 Chair and Department of Human Physiology, Medical University of Lublin, 20-080 Lublin, Poland; weronikakasprzycka2@gmail.com

**Keywords:** cellular immunology, Extended Inflammation Parameters (EIP), activated lymphocytes, neutrophil activation parameters

## Abstract

(1) Background: Pemphigus is a blistering autoimmune disease of the skin and/or mucous membranes, characterised by the presence of specific autoantibodies directed against structural proteins of the human skin. Recent reports indicate that new haematological parameters, termed Extended Inflammation Parameters (EIP), can be used to assess the activation of immune cells during active inflammation. These include parameters assessing both neutrophil activation (NEUT-RI, NEUT-GI) and the number of activated lymphocytes (RE-LYMP). The aim of this study was to investigate the relationship between changes in NEUT-RI, NEUT-GI and RE-LYMP and the disease activity in patients with pemphigus. (2) Results: The study involved 32 patients with diagnosed different types of pemphigus. Neutrophil activation parameters (NEUT-RI and NEUT-GI) and lymphocytes (RE-LYMP) were significantly higher in these patients compared to the parameters in healthy participants (respectively *p* = 0.0127, *p* = 0.0011 and *p* = 0.0033). The increased quantity of activated lymphocytes (RE-LYMP) also correlated significantly with the extent of skin and/or mucosal lesions in patients assessed by the PDAI scale (*p* < 0.02). (3) Conclusions: The NEUT-RI, NEUT-GI and RE-LYMP parameters proved to be appropriate markers of inflammation severity in pemphigus, also in relation to local lesions, which was not possible with the inflammation markers (CRP, ESR) used so far on a routine basis.

## 1. Introduction

Pemphigus is a blistering autoimmune disease of the skin and/or mucous membranes that is characterised by the presence of specific autoantibodies directed against structural proteins of the human skin [1]. The autoantigens to which autoantibodies are directed are various desmosomal adhesion molecules [2]. The presence of autoantibodies in the patient’s circulation or in the intercellular spaces of the epidermis or epithelium leads to acantholysis and the appearance of blisters and erosions on the skin and/or mucous membranes [3,4].

Pemphigus is not a hereditary disease. Its form depends on which antigen is attacked by the autoantibodies. Making a definitive diagnosis requires, firstly, a thorough medical history, including the course of the disease, accompanying symptoms and co-morbidities or medication taken [5,6]. The second stage of diagnosis should include an assessment of the extent of skin and mucosal lesions according to the Pemphigus Disease Area Index (PDAI) scale [7], the severity of the so-called Nikolsky’s sign [8] and the patient’s general condition, including the assessment of internal organ dysfunction. The diagnosis should be confirmed and appropriate treatment can be administered by laboratory tests: direct immunopathological examination of a skin section by direct immunofluorescence (DIF), examination of the patient’s serum for specific antibodies by indirect immunofluorescence (IIF), examination for the presence of autoantibodies by immunoenzymatic methods (enzyme-linked immunosorbent assay-ELISA, immunoblot-IB, immunoprecipitation-IP) and routine histopathological examination [9]. The presence of autoantibody deposits in the intercellular spaces of the epidermis or epithelium, unbound (free) autoantibodies in the patient’s blood serum or acantholysis and/or neutrophilic infiltrates are indicative of a particular type of pemphigus, while also allowing for differentiation from other blistering skin diseases such as pemphigoid [10].

Based on the above-mentioned clinical and laboratory information, several clinical variants of pemphigus can be distinguished. Types of pemphigus differ in terms of antibodies that are directed against other structures of the epidermis. The result is a different clinical course.

Pemphigus is associated with numerous discomforts and reduces quality of life. Unfortunately, it is a multifaceted disease whose laboratory criteria, although based on specialized and costly methods, do not always provide sufficient information about the disease’s course. The search for new markers of severity of inflammation is therefore reasonable [11].

**Figure 1:**
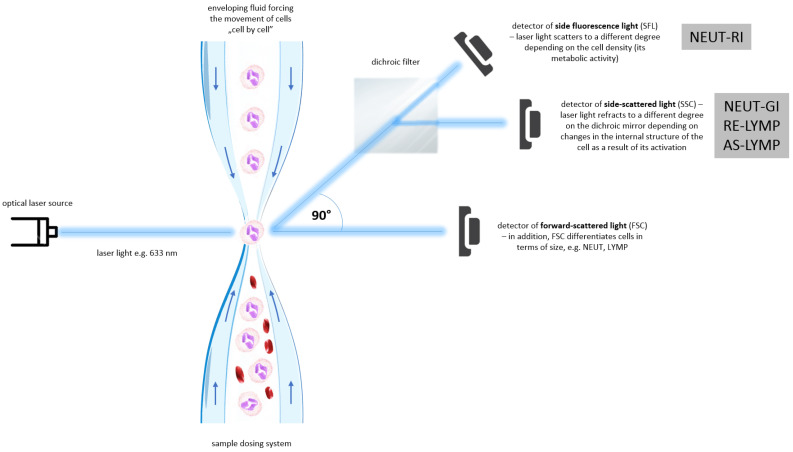
Extended Inflammation Parameters (EIP) measurement method. See references in main text. (AS-LYMP–Antibody-Secreting Reactive Lymphocytes, FSC–Forward-Scattered Light, LYMP–Lymphocytes, NEUT–Neutrophils, NEUT-GI–Neutrophil Granularity Intensity, NEUT-RI–Neutrophil Reactive Intensity, RE-LYMP–Reactive Lymphocytes, SFL–Side Fluorescence Light, SSC–Side-Scattered Light).

This study focuses on the evaluation of new hematological markers of inflammation-Extended Inflammation Parameters (EIP) in pemphigus. The evaluated parameters include NEUT-RI (Neutrophil Reactive Intensity, expressed in FI units, showing fluorescence intensity), NEUT-GI (Neutrophil Granularity Intensity, expressed in SI units, showing laser light scattering intensity), RE-LYMP (Reactive Lymphocytes, expressed as the number of cells per µL) and AS-LYMP (Antibody-Secreting Reactive Lymphocytes, expressed as the number of cells per µL) [12]. RE-LYMP parameter provides information on the amount of all reactive lymphocytes in peripheral blood. Lymphocyte populations are differentiated on the basis of their functionality and differences resulting from their functionality in internal structure, present granularity and size of the analysed cells which is accessible in SYSMEX XN haematological analysers. The neutrophil parameters, i.e., NEUT-RI and NEUT-GI inform about the stage of activation of neutrophil granulocytes. The measurement considers the metabolic activity of neutrophils, their internal structure and cell size [13]. EIP measurement method was shown in Figure 1.

**Figure 2:**
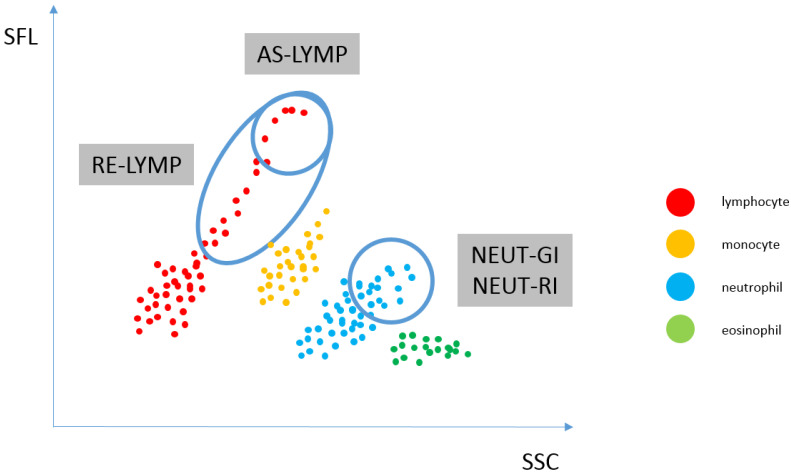
Reflecting the amount of reactive lymphocytes and neutrophils at different activation stages in the peripheral blood fraction. WDF scattergram showing the distribution of populations: lymphocytes (AS-LYMP population–upper marking and RE-LYMP population–lower marking), monocytes, neutrophils [the SSC signal of the neutrophil population, which is plotted on the x-axis of the scattergram, is an indicator of the granularity and internal structure of the cells (NEUT-GI) and the fluorescence intensity, which corresponds to the RNA/DNA content of the cells, is plotted on the y-axis and is an indicator of increased RNA activity (NEUT-RI)] as well as eosinophils and basophils. (AS-LYMP–Antibody-Secreting Reactive Lymphocytes, NEUT-GI–Neutrophil Granularity Intensity, NEUT-RI–Neutrophil Reactive Intensity, RE-LYMP–Reactive Lymphocytes).

By reflecting the amount of reactive lymphocytes and neutrophils at different activation stages in the fraction peripheral blood, inflammation can be distinguished from infection and even the causative agent of infection (viral or bacterial) can be distinguished from the type of immune response (innate or adaptive, cellular or humoral). When neutrophils and lymphocytes are activated with various intracellular pathways of inflammatory response, the levels of the EIP parameters (RE-LYMP, NEUT-RI, NEUT-GI) also increase. The change in these parameters depends on the nature of the inflammatory stimulus, severity and stage of the infection [14]. The amount of reactive lymphocytes and neutrophils at different activation stages in the peripheral blood fraction is shown in Figure 2.

Our research, discussed in our previous article [12], clearly shows that an EIP-enriched CBC study in people with psoriasis can help improve the diagnostic interpretation of patient test results. It was decided to make similar observations for pemphigus. It should be emphasised that these parameters have never previously been evaluated for their diagnostic utility in relation to this disease. As a result, they may be useful in assessing immune cell activation on a daily basis as the disease progresses.

## 2. Materials and Methods

### 2.1. Characteristics of Patients

32 patients from the Department of Dermatology, Venereology and Paediatric Dermatology of the Independent Public Clinical Hospital No. 1 in Lublin were included in the study. 16 women and 16 men aged over 18 years (mean age amounted to 58 years) were eligible for inclusion in the study. Patients diagnosed with various types of pemphigus at different stages of treatment (see Table 1, column 3 for details) met the inclusion criteria. Healthy participants made up the control group, which consisted of 32 volunteers over the age of 18. The absolute inclusion criteria for this group were the absence of ongoing inflammation and normal results of common laboratory tests (see Table 1, column 4 for details). All participants had 10 mL of venous blood collected using disposable equipment. Blood concentrations of selected haematological and biochemical parameters were then determined within 2 h of collection. The Bioethics Committee at the Medical University of Lublin approved the study and granted permission for its implementation with Resolution no. KE-0254/232/2019.

### 2.2. Apparatus and Methodology

Serum and whole blood drawn from the ulnar vein were used for the study. Fasting patients provided the material, which was collected in the morning. First, blood samples (approx. 7.6 mL each) were collected and placed in vacutainer serum clot activator tubes for biochemical determinations. The samples were then collected into 2.7 mL vacutainer tubes containing the K3EDTA (ethylenediaminetetraacetic acid tripotassium salt) anticoagulant to obtain whole blood and determine the selected haematological parameters. They underwent an analysis within 2 h of being collected.

Haematological determinations were carried out using a Sysmex XN 1500 apparatus (Sysmex Europe SE, Warsaw, Poland) and an Alifax Roller 20PN apparatus (Alifax S.r.l. Warsaw, Poland), and biochemical determinations were made with a COBAS 6000 analyser (Roche Diagnostics, Warsaw, Poland).

The examined parameters include: Neutrophil Reactive Intensity (NEUT-RI), Neutrophil Granularity Intensity (NEUT-GI), Antibody-Secreting Reactive Lymphocytes (AS-LYMP), Reactive Lymphocytes (RE-LYMP), White Blood Cells (WBC), Neutrophils (NEUT), Lymphocytes (LYMP), Monocytes (MONO), Eosinophils (EO), Basophils (BAZO), Immature Granulocyte Count (IG), Erythrocytes (RBC), Hemoglobin (HGB), Hematocrit (HCT), Mean Cell Hemoglobin (MCH), Mean Corpuscular Hemoglobin Concentration (MCHC), Mean Corpuscular Volume (MCV), Platelets (PLT), total bilirubin, total protein, Aspartate Aminotransferase (AST), Alanine Aminotransferase (ALT), C-reactive protein (CRP), glucose and Erythrocyte Sedimentation Rate (ESR).

#### Measurement of EIP Parameters on an XN-Series Haematology Analyser

The blood sample is first dispensed and aspirated through a dispensing system, after which it is diluted and labelled with a fluorescent marker that binds specifically to nucleic acids. The sample is then transported to a flow cell and illuminated by a solid-state laser beam that penetrates the cells with a wavelength of 663 nm. The appearance of a cell or molecule in the path of the laser beam results in three different signals: side fluorescence light (SFL), forward-scattered light (FSC) and side-scattered light (SSC). FSC provides information about the cell volume, SSC about the internal structure of the cell (nucleus and granules), while the intensity of SFL indicates the amount of nucleic acids and cell organelles present in the analysed morphotic element.

Differentiation and counting of neutrophils, lymphocytes, monocytes and eosinophils takes place in the WDF (white differential channel). Surfactants in the reagent cause haemolysis of red blood cells, dissolution of platelets and formation of perforations in the cell membrane of white blood cells. Particular subpopulations of white cells are distinguished using the SSC detector. A fluorescent dye then enters the cell cavity and stains the nucleic acids as well as cell organelles. The intensity of the SFL signal depends on white blood cell type, size, nucleic acid content and cell organelles.

Positioning of neutrophil and lymphocyte populations on the WDF scattergram, allows for the assessment of neutrophil and reactive lymphocyte activation. By analysing the cloud and differences in diffused and fluorescent light, it is possible to differentiate and count cells. Activated cells, i.e., neutrophils and lymphocytes, show a greater fluorescence signal than resting cells. The NEUT-RI parameter reflects the intensity of neutrophil reactivity depending on their metabolic activity, while the NEUT-GI parameter will provide information on cell density and granularity. The AS-LYMP parameter represented as lymphocytes with the highest fluorescence signal reflects activated B lymphocytes that produces antibodies. The RE-LYMP parameter represented as lymphocytes having a higher fluorescence signal than the physiological lymphocyte population, reflects reactive lymphocytes (see Figure 1 and Figure 2).

### 2.3. Statistical Methods

StatSoft’s Statistica 13 software was used to conduct statistical analysis of the study results. The Student’s t-distribution and the ANOVA test were applied to determine statistical significance. The 5% margin of error was allowed and the statistical significance level was set at *p* < 0.05, indicating the presence of statistically significant differences or dependencies.

## 3. Results

The results obtained during the study were analysed. 25 haematological and biochemical parameters were considered: (1) NEUT-RI [FI], (2) NEUT-GI [SI], (3) AS-LYMP [10^3^/µL], (4) RE-LYMP [10^3^/µL], (5) WBC [K/µL], (6) NEUT [K/µL], (7) LYMP [K/µL], (8) MONO [K/µL], (9) EO [K/µL], (10) BAZO [K/µL], (11) GI [K/µL], (12) RBC [M/µL], (13) HGB [g/dL], (14) HCT [%], (15) MCH [pg], (16) MCHC [g/dL], (17) MCV [fL], (18) PLT [K/µL], (19) total bilirubin [mg/dL], (20) total protein [g/dL], (21) AST [IU/L], (22) ALT [U/L], (23) CRP [mg/L], (24) glucose [mg/dL], (25) ESR [mm/h]. These parameters were statistically characterised for 32 patients with pemphigus (Study group) and 32 healthy controls (Control group). The results obtained in the analyses are presented in Table 2.

Analysis of descriptors of white blood cells showed that in patients with pemphigus (irrespective of its variant), the levels of parameters assessing neutrophil activation, i.e., NEUT-RI and NEUT-GI, were significantly higher compared to the levels of these parameters in healthy participants (*p* = 0.0127 and *p* = 0.0011, respectively, Table 2 lines 1–2). A statistically significant difference was also observed for the level of the parameter assessing RE-LYMP lymphocyte activation (Table 2, line 4). RE-LYMP values were significantly higher in the pemphigus group compared to the control group (*p* = 0.0033).

Statistically significant differences were also found when assessing the total white blood cell count (Table 2 line 5), neutrophil count (Table 2 line 6), monocyte count (Table 2 line 8), eosinophil count (Table 2 line 9) and immature granulocyte count (Table 2 line 11). Patients with pemphigus have significantly higher levels of these parameters than their healthy controls (*p* = 0.0121, *p* = 0.0020, *p* = 0.0001, *p* = 0.0167 and *p* = 0.0019 respectively). There was also a statistically significant difference in erythrocyte sedimentation rate in the group of patients with pemphigus (Table 2 line 25), compared to the control group (*p* = 0.0031). These values were significantly higher in patients with pemphigus compared to healthy participants. There was also a statistically significant reduction in mean corpuscular haemoglobin concentration (MCHC) in pemphigus patients (Table 2 line 16). Pemphigus patients showed significant changes in total bilirubin levels (Table 2 line 19). These are unexpected incidental findings that are not related to immune cell activation. They may have their origin in associated diseases and require further investigation. No statistically significant differences were found between the compared groups of patients for the other parameters.

Parameters for which significant differences were observed between pemphigus patients and controls, were analyzed in terms of pemphigus type including: (1) 22 patients with pemphigus vulgaris (PV), (2) 4 patients with pemphigus foliaceus (PF), (3) 2 patients with pemphigus vegetans (PG), (4) 3 patients with pemphigus herpetiformis (PH) and (5) 1 patient with pemphigus erythematosus.

Statistically significant differences in NEUT-RI levels were observed in patients diagnosed with pemphigus vegetans (*p* = 0.0206), who had higher NEUT-RI results (Table 3, line 1). Similar data were received for NEUT-GI in patients diagnosed with pemphigus vulgaris (*p* = 0.0405), who had higher NEUT-GI results in comparison with the other pemphigus variants analyzed (Table 3, line 2). When comparing the levels of NEUT-RI and NEUT-GI in the other pemphigus types, as well as the RE-LYMP parameter, no statistically significant differences were noted. In addition, patients with pemphigus vegetans had higher MONO (*p* = 0.0194) results than pemphigus vulgaris (Table 3, line 6). Patients with pemphigus herpetiformis had higher EO results (*p* = 0.0243) (Table 3, line 7) and patients with pemphigus erythematosus had higher IG results (*p* = 0.0047) than in pemphigus foliaceus (Table 3, line 8). For the other WBC parameters, there were no statistically significant differences according to the type of pemphigus diagnosed. The analysis of the other parameters showed that the pemphigus variant had no effect on their values.

The laboratory results obtained were also analyzed in terms of the duration of pemphigus in the patient. There was a statistically significant increase in WBC values (*p* = 0.0275), the longer the disease lasted.

In addition, biochemical and haematological parameters were compared in patients with a various range of skin and/or mucosal lesions (according to the PDAI score) during active pemphigus and its absence. Statistically significant differences in RE-LYMP values were observed according to the extent of skin and/or mucosal lesions during pemphigus activity (*p* = 0.02) (see Table 4, line 3). The more points a patient scored on the PDAI scale, the higher the numbers of activated lymphocytes were observed. For the other parameters, including the extent of skin and/or mucosal lesions when the pemphigus resolved, there were no statistically significant relationships.

The biochemical and haematological parameters analysed were compared to results obtained by direct immunofluorescence (DIF) and indirect immunofluorescence (IIF). No statistically significant relationships have been noted. However, it is noteworthy that the RE-LYMP parameter, indicative of lymphocyte activation, tended to increase with the growth of IIF autoantibody titres (*p* = 0.065).

The values of biochemical and haematological parameters in patients according to the implementation of treatment or no treatment were also analyzed. It is worth mentioning that when systemic steroid therapy or azathioprine treatment was implemented, a significant increase in total bilirubin values was observed (*p* = 0.0203 and *p* = 0.0041, respectively). These changes did not appear in patients treated with intravenous immunoglobulin (IVIG), dapsone and rituximab.

## 4. Discussion

Pemphigus is an autoimmune disease in which a strongly marked inflammation, mainly of a local nature, is frequently observed [15]. In laboratory terms, this is often manifested by accelerated ESR [16] or elevated levels of inflammatory parameters [17]. Our study also showed a definite correlation of ESR acceleration and increases in total white blood cell count and individual white blood cell fractions with the severity of inflammation in pemphigus. Due to minor differences in the pathomechanism of pemphigus variants, discrepancies in laboratory tests are also observed, as exemplified by the notable monocytosis and eosinophilia in pemphigus vegetans. However, it should be emphasized that activation of the immune system, manifested primarily by increased neutrophil [18] and lymphocyte [19,20], is significant in patients diagnosed with pemphigus. It is therefore essential that study is carried out to understand as precisely as possible the mechanism of immune cell activation in the course of this disease.

**Figure 3:**
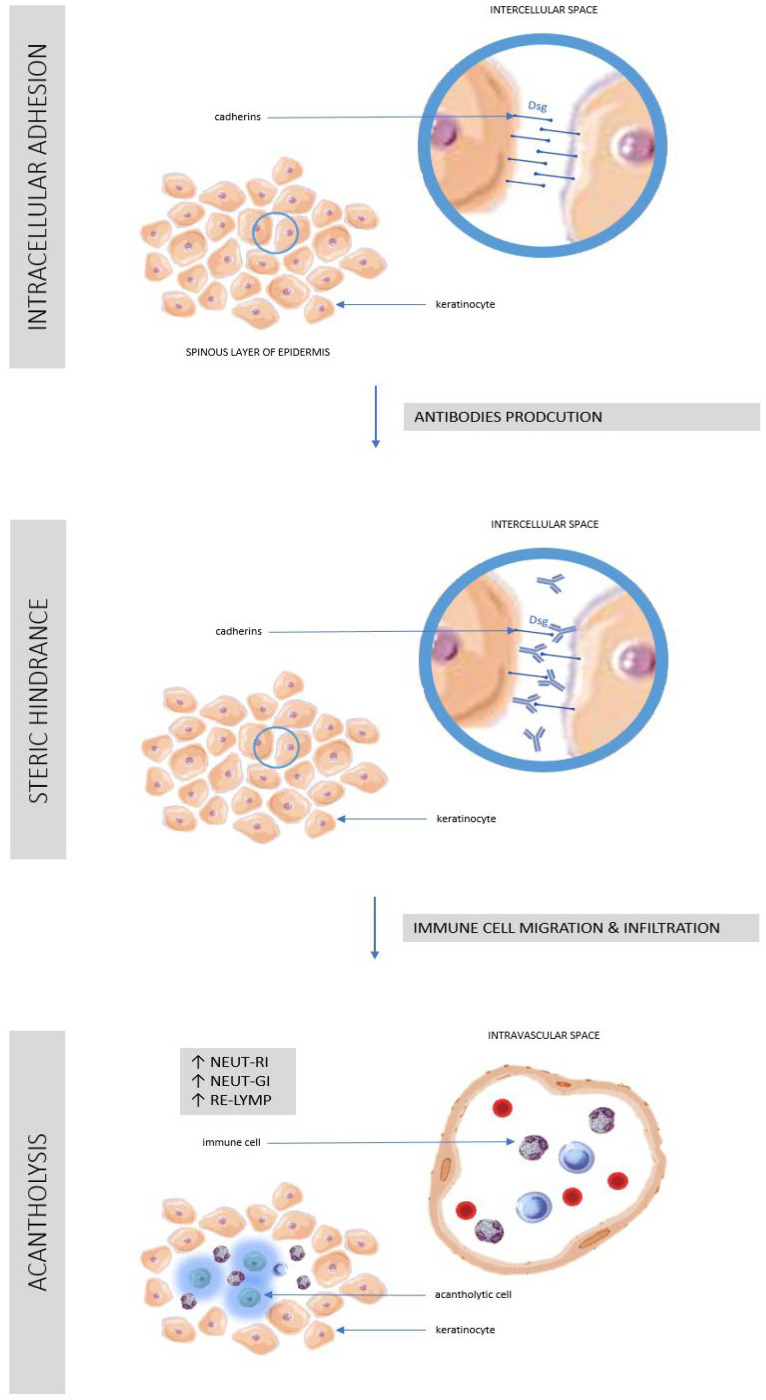
Pathophysiology of pemphigus and the role of immune cells in the development of lesions. See references in main text. (AS-LYMP–Antibody-Secreting Reactive Lymphocytes, NEUT-GI–Neutrophil Granularity Intensity, NEUT-RI–Neutrophil Reactive Intensity, RE-LYMP–Reactive Lymphocytes).

It is known that in healthy people about half the population of white blood cells in the blood circulation are neutrophils, also known as neutrophil granulocytes. These are phagocytes that are part of primary immunity [21]. Following an inflammatory stimulus, changes in neutrophil morphology and motility occur due to activation of different immune response strategies. Depending on the trigger of the inflammatory process, neutrophils may use phagocytosis, the secretion of a number of pro-inflammatory cytokines or the release of Neutrophil Extracellular Traps (NET) [22]. Pathophysiology of pemphigus and the role of immune cells in the development of lesions has been shown in Figure 3.

Confirmation of this neutrophil activation in pemphigus is provided by the results of our analysis showing an increase in the NEUT-RI and NEUT-GI descriptors in patients with different types of this disease. Neutrophil activation probably results mainly from the action of IL-17 (interleukin 17) and IL-36 (interleukin 36) on these cells [23]. Neutrophil activation is clinically manifested by neutrophil infiltration of blisters formed during the course of the disease. This is evident in the histological examination of skin sections of affected individuals and translates directly into increased local inflammation in the regions of skin lesions [24]. An important finding is that NEUT-RI is significantly increased in patients with pemphigus vegetans and NEUT-GI is increased in patients with pemphigus vulgaris compared to both healthy individuals and other types of pemphigus. The NEUT-RI reflects the severity of neutrophil reactivity expressed by fluorescence intensity (FI) [25]. Higher values of this descriptor in patients diagnosed with pemphigus vegetans indicate greater neutrophil metabolic activity in this type of pemphigus. This allows us to conclude that, among other things, the significant activation of a number of intracellular enzymes in neutrophils leads to the production of reactive oxygen species and the release of oxygen free radicals (the so-called oxidative burst). This mechanism probably greatly enhances the formation of new inflammatory foci in pemphigus vegetans. The NEUT-GI parameter, which increases more in patients with pemphigus vulgaris, expressed as laser scattering intensity (SI), provides information about the internal structure of neutrophils [26]. This may indicate that strategies based on neutrophil degranulation are more prominent in pemphigus vulgaris. The intracellular structure of neutrophils may be altered due to the mobilization of secretory vesicles by the action of inflammatory mediators present in the patient’s serum.

Similar observations were made when analyzing a haematological parameter indicative of lymphocyte activation, the RE-LYMP. In patients with pemphigus, the increase in activated lymphocytes is primarily associated with their involvement in the production of pathogenic autoantibodies, which in turn induce intraepidermal blister formation [27]. However, this is not their only role. Lymphocytes also create cellular infiltrates at sites of bullous lesions. They thus modulate local inflammation by, among other things, the cytokines they produce. The relationship between the severity and extent of skin and/or mucosal lesions, as assessed by the PDAI scale, and the level of the RE-LYMP parameter, both its absolute value and percentage, is therefore understandable. The number of activated lymphocytes directly translates into inflammatory activity at the site of blistering lesions. This in turn affects their severity and extent. The involvement of T lymphocytes in the pathogenesis of skin and/or mucosal lesions in pemphigus was studied as early as 2013 [28]. The results obtained by our team confirm these reports.

Lyakhovitsky and colleagues [29] in a recent paper also confirmed the validity of neutrophil and lymphocyte counts in pemphigus patients. Admittedly, their study was based on assessing the neutrophil-to-lymphocyte ratio, platelet-to-lymphocyte ratio, platelet-to-neutrophil ratio and mean platelet volume, without addressing the activation of these cells during the course of the disease. Our study, in conjunction with the findings of the Lyakhovitsky research group, allows us to conclude that changes in the number of individual fractions of white blood cells during pemphigus activation are due precisely to the stimulation of immunocompetent cells during inflammation. This thus confirms the usefulness of the NEUT-RI, NEUT-GI and RE-LYMP parameters in the assessing of the severity of pemphigus.

The obtained results are not without limitations. The basic limitations include: (1) a small research group, (2) the possibility of additional undiagnosed conditions causing inflammation in the patient, (3) laboratory and per-laboratory bias.

## 5. Conclusions

In conclusion, the analysis of immune cell activation parameters in pemphigus is extremely important. The NEUT-RI, NEUT-GI and RE-LYMP parameters have proven to be excellent markers of the severity of inflammation in this disease entity, also in relation to local lesions, which is not possible with the inflammatory markers routinely used to date (CRP, ESR). The results of our study may suggest the usefulness of EIP parameters as the markers of neutrophil and lymphocyte activation for assessing the severity of pemphigus. Further studies are needed to establish the role of EIP parameters in pemphigus, especially in the monitoring of the disease.

## Figures and Tables

**Table 1:** Characteristics of the study group and the control group.

		Total N = 64 Range/(Average/%)	
	**Demographic Data**		
		**Study Group = 32**	**Control Group = 32**
1.	Age	33–94 (58.00)	21–90 (57.00)
2.	Male participants	16 (50.00)	14 (43.75)
	**Clinical Data**		
3.	Pemphigus vulgaris (PV)	22 (68.75)	0
4.	Pemphigus foliaceus (PF)	4 (12.5)	0
5.	Pemphigus vegetans (PG)	2 (6.25)	0
6.	Pemphigus herpetiformis (PH)	3 (9.38)	0
7.	Pemphigus erythematosus (PE)	1 (3.13)	0
8.	Duration of disease (years)	0.1–24.0 (4.04)	-
9.	Positive test result by: • DIF • IIF	21 (65.63) 24 (75.00)	0 0
10.	Accompanying diseases ^1^	26 (81.25)	21 (65.63)
11.	Tumours	1 (3.13)	0
12.	Treatment administered before the study: Systemic steroid therapy Azathioprine IVIG DapsonRituximab	20 (62.50) 15 (46.88) 1 (3.13) 1 (3.13) 2 (6.25)	2 (6.25) 0 0 0 0
13.	Current treatment: Systemic steroid therapy Azathioprine IVIG Dapson Rituximab	22 (68.75) 13 (40.63) 0 1 (3.13) 2 (6.25)	0 0 0 0 0

^1^ including type 2 diabetes (T2D), hypertension, iron deficiency anaemia, hyperthyroidism, status post melanoma removal, Warthin’s tumour, gastritis, myasthenia gravis, allergic rhinitis, impaired glucose tolerance, obesity, schizophrenia, osteoarthritis, NAFLD, hypercholesterolemia, insulin resistance, PCOS, chronic venous insufficiency, gastroesophageal reflux disease, coronary artery disease, hypothyroidism, spinal osteoarthritis, cholecystolithiasis, prostate growth, scabies, status post myocardial infarction, rheumatoid arthritis, uterine myoma, ovarian cyst, migraine, reactive hypoglycaemia, nodular thyroid goitre, atrial fibrillation. * DIF–Direct Immunofluorescence, IIF–Indirect Immunofluorescence, IVIG–Intravenous Immune Globulin N–the number of all variables, NAFLD–nonalcoholic fatty liver disease, PCOS–polycystic ovary syndrome, PE–pemphigus erythematosus, PF–pemphigus foliaceus, PG–pemphigus vegetans, PH–pemphigus herpetiformis, PV–pemphigus vulgaris.

**Table 2:** Statistical characteristics of biochemical and haematological parameters in patients with pemphigus (GP) and control group (GK).

	Parameter	Study Group (N = 32)					Control Group (N = 32)					*p*
		Av	SD	Me	25–75% Percentile	Min–Max	Av	SD	Me	25–75% Percentile	Min–Max	
1.	NEUT-RI [FI]	47.09	4.80	46.35	43.75–49.50	41.60–64.60	44.19	2.20	43.50	42.70–45.25	40.30–50.10	**0.0127**
2.	NEUT-GI [SI]	150.84	4.49	150.70	147.40–154.60	141.30–159.40	147.04	4.43	146.85	143.70–150.00	137.80–157.00	**0.0011**
3.	AS-LYMP [10^3^/µL]	0.01	0.00	0.01	0.00–0.00	0.00–0.06	0.00	0.00	0.00	0.00–0.00	0.00–0.00	0.3251
4.	RE-LYMP [10^3^/µL]	0.11	0.08	0.07	0.06–0.15	0.02–0.37	0.04	0.02	0.03	0.02–0.04	0.01–0.08	**0.0033**
5.	WBC [K/µL]	7.62	2.14	7.46	5.84–9.39	3.84–11.98	6.01	1.38	5.96	5.14–6.91	3.44–9.34	**0.0121**
6.	NEUT [K/µL]	4.40	1.38	4.15	3.53–5.68	1.81–6.88	3.38	1.15	3.40	2.35–4.12	1.56–5.59	**0.0020**
7.	LYMP [K/µL]	2.21	0.79	2.22	1.72–2.64	0.71–4.18	1.94	0.48	1.82	1.61–2.32	1.82–2.92	0.1063
8.	MONO [K/µL]	0.71	0.22	0.68	0.54–0.87	0.35–1.18	0.52	0.14	0.49	0.40–0.61	0.31–0.78	**0.0001**
9.	EO [K/µL]	0.26	0.23	0.19	0.11–0.35	0.00–0.91	0.15	0.11	0.16	0.07–0.20	0.02–0.47	**0.0167**
10.	BAZO [K/µL]	0.04	0.03	0.04	0.03–0.05	0.00–0.11	0.03	0.01	0.03	0.02–0.04	0.01–0.07	0.2337
11.	IG [K/µL]	0.04	0.02	0.03	0.02–0.05	0.01–0.10	0.02	0.02	0.02	0.01–0.02	0.00–0.08	**0.0019**
12.	RBC [M/µL]	4.55	0.66	4.64	4.22–5.00	2.51–5.89	4.67	0.47	4.66	4.29–5.03	3.57–5.72	0.3637
13.	HGB [g/dL]	13.47	2.16	13.65	12.50–15.20	7.00–16.40	13.85	1.26	13.55	12.85–14.85	11.90–16.40	0.3959
14.	HCT [%]	39.92	5.61	40.55	36.95–44.25	21.50–47.00	40.51	3.41	40.05	37.70–43.25	34.40–48.30	0.6182
15.	MCH [pg]	29.63	2.06	30.10	28.95–30.90	23.60–32.90	29.69	1.59	29.50	28.55–30.55	27.00–34.70	0.8923
16.	MCHC [g/dL]	33.65	1.20	33.75	32.75–34.50	31.10–36.20	34.18	0.76	33.95	33.60–34.75	33.10–36.00	**0.0372**
17.	MCV [fL]	88.02	4.60	88.60	86.30–91.20	76.10–94.90	86.83	3.60	86.55	84.75–88.40	79.20–96.40	0.2519
18.	PLT [K/µL]	254.78	54.62	254.00	207.50–290.50	143.00–369.00	244.03	68.11	243.50	196.50–287.00	103.00–394.00	0.4887
19.	Total bilirubin [mg/dL]	0.47	0.19	0.43	0.30–0.65	0.19–0.80	0.79	0.37	0.67	0.57–0.90	0.35–1.68	**0.0036**
20.	Total protein [g/dL]	6.79	0.54	7.01	6.26–7.16	5.87–7.43	7.08	0.56	7.18	6.89–7.43	5.59–7.93	0.1975
21.	AST [IU/L]	18.73	6.29	18.20	13.90–22.00	9.80–35.60	22.34	10.88	19.00	16.50–24.30	11.20–64.40	0.1112
22.	ALT [U/L]	22.57	11.82	22.35	13.70–30.55	5.00–50.90	19.57	11.84	15.80	11.80–25.60	6.20–63.70	0.3196
23.	CRP [mg/L]	14.57	31.36	2.30	1.15–10.45	0.60–120.60	2.54	2.57	1.20	1.00–3.30	1.00–8.90	0.1029
24.	Glucose [mg/dL]	95.55	45.29	89.10	81.30–93.70	24.70–315.40	91.35	9.11	88.40	84.30–95.50	81.20–121.50	0.6095
25.	ESR [mm/h]	25.00	23.08	18.00	10.00–34.00	2.00–89.00	10.22	6.68	9.50	4.50–12.50	2.00–27.00	**0.0031**

* ALT–Alanine Aminotransferase, AST–Aspartate Aminotransferase, AS-LYMP–Antibody-Secreting Reactive Lymphocytes, Av–average, BAZO–Basophils, CRP–C-reactive protein, EO–Eosinophils, ESR–Erythrocyte Sedimentation Rate, HCT–Hematocrit, HGB–Hemoglobin, IG–Immature Granulocyte Count, LYMP–Lymphocytes, MCH–Mean Cell Hemoglobin, MCHC–Mean Corpuscular Hemoglobin Concentration, MCV–Mean Corpuscular Volume, Me–Median, MONO–Monocytes, N–the number of all variables, NEUT–Neutrophils, NEUT-GI–Neutrophil Granularity Intensity, NEUT-RI–Neutrophil Reactive Intensity, PLT–Platelets, RBC–Erythrocytes, RE-LYMP–Reactive Lymphocytes, SD–Standard Deviation, WBC–White Blood Cells.

**Table 3:** Dependence of selected parameters on the type of pemphigus.

	Parameter	Type of Pemphigus	Av	SD	Me	*p*
1.	**NEUT-RI [FI]**	PV	47.00	5.19	46.70	**0.0206**
		PF	45.70	4.22	44.95	
		**PG**	**47.85**	**1.48**	**47.85**	
		PH	42.83	1.07	42.60	
		PE	44.10	-	-	
2.	**NEUT-GI [SI]**	**PV**	**158.09**	**3.87**	**156.65**	**0.0405**
		PF	153.30	5.61	153.43	
		PG	154.10	0.71	154.10	
		PH	145.80	4.83	145.20	
		PE	144.10	-	-	
3.	RE-LYMP [10^3^/µL]	PV	0.12	0.09	0.08	0.4029
		PF	0.08	0.06	0.05	
		PG	0.10	0.04	0.10	
		PH	0.08	0.04	0.06	
		PE	0.06	-	-	
4.	WBC [K/µL]	PV	7.28	1.99	7.42	0.5241
		PF	8.60	3.34	8.39	
		PG	9.16	2.36	9.16	
		PH	7.79	2.17	6.66	
		PE	7.57	-	-	
5.	NEUT [K/µL]	PV	4.25	1.29	4.09	0.3981
		PF	4.91	2.04	5.06	
		PG	5.30	1.60	5.30	
		PH	4.09	1.66	3.44	
		PE	4.75	-	-	
6.	**MONO [K/µL]**	PV	0.67	0.20	0.66	**0.0194**
		PF	0.85	0.35	0.85	
		**PG**	**0.92**	**0.00**	**0.92**	
		PH	0.60	0.09	0.63	
		PE	0.85	-	-	
7.	**EO [K/µL]**	PV	0.22	0.19	0.17	**0.0243**
		PF	0.21	0.15	0.16	
		PG	0.52	0.42	0.52	
		**PH**	**0.61**	**0.23**	**0.59**	
		PE	0.26	-	-	
8.	**IG [K/µL]**	PV	0.04	0.02	0.03	**0.0047**
		PF	0.03	0.02	0.03	
		PG	0.04	0.03	0.04	
		PH	0.02	0.02	0.01	
		**PE**	**0.06**	-	-	
9.	MCHC [g/dL]	PV	33.53	1.13	33.70	0.4453
		PF	34.33	1.56	34.35	
		PG	33.10	1.98	33.10	
		PH	33.50	1.00	33.50	
		PE	35.00	-	-	
10.	Total bilirubin [mg/dL]	PV	0.45	0.19	0.44	0.1030
		PF	0.41	0.10	0.44	
		PG	0.42	0.01	0.42	
		PH	0.48	0.10	0.44	
		PE	0.42	-	-	
11.	ESR [mm/h]	PV	29.28	24.21	25.50	0.5987
		PF	12.33	9.29	15.00	
		PG	34.50	43.13	34.50	
		PH	11.67	1.53	12.00	
		PE	7.00	-	-	

* Av–average, EO–Eosinophils, ESR–Erythrocyte Sedimentation Rate, IG–Immature Granulocyte Count, MCHC–Mean Corpuscular Haemoglobin Concentration, Me–Median, MONO–Monocytes, NEUT–Neutrophils, NEUT-GI–Neutrophil Granularity Intensity, NEUT-RI–Neutrophil Reactive Intensity, PE–pemphigus erythematosus, PF–pemphigus foliaceus, PG–pemphigus vegetans, PH–pemphigus herpetiformis, PV–pemphigus vulgaris, RE-LYMP–Reactive Lymphocytes, SD–Standard Deviation, WBC–White Blood Cells.

**Table 4:** Dependence of selected parameters on the extent of skin and/or mucosal lesions (according to the PDAI scale) during pemphigus activity.

	Parameter	Group of PDAI Scale	Av	SD	Me	*p*
1.	NEUT-RI [FI]	0–6	46.14	3.57	44.90	0.2677
		7–13	46.17	4.48	44.60	
		14–20	47.45	1.63	47.45	
		21–27	49.50	0.99	49.50	
		28–34	51.67	11.22	45.80	
2.	NEUT-GI [SI]	0–6	150.22	3.99	149.70	0.8161
		7–13	151.42	5.63	150.80	
		14–20	146.55	3.04	146.55	
		21–27	151.00	0.71	151.00	
		28–34	154.70	1.65	154.60	
**3.**	**RE-LYMP [10^3^/µL]**	**0–6**	**0.04**	**0.01**	**0.03**	**0.0202**
		**7–13**	**0.11**	**0.07**	**0.09**	
		**14–20**	**0.12**	**0.05**	**0.12**	
		**21–27**	**0.15**	**0.08**	**0.15**	
		**28–34**	**0.19**	**0.05**	**0.17**	
4.	WBC [K/µL]	0–6	7.13	1.63	6.75	0.3235
		7–13	8.10	2.34	7.42	
		14–20	7.18	4.72	7.18	
		21–27	7.85	2.17	7.85	
		28–34	7.84	2.20	8.79	
5.	NEUT [K/µL]	0–6	4.19	1.27	3.99	0.3881
		7–13	4.54	1.53	4.40	
		14–20	3.94	2.40	3.94	
		21–27	5.07	1.44	5.07	
		28–34	4.41	0.92	4.13	
6.	MONO [K/µL]	0–6	0.68	0.18	0.66	0.1981
		7–13	0.72	0.24	0.67	
		14–20	0.72	0.52	0.72	
		21–27	0.76	0.36	0.76	
		28–34	0.74	0.12	0.76	
7.	EO [K/µL]	0–6	0.23	0.26	0.12	0.4159
		7–13	0.30	0.19	0.24	
		14–20	0.44	0.48	0.44	
		21–27	0.12	0.02	0.12	
		28–34	0.27	0.11	0.29	
8.	IG [K/µL]	0–6	0.03	0.02	0.03	0.4778
		7–13	0.04	0.03	0.04	
		14–20	0.03	0.01	0.03	
		21–27	0.03	0.01	0.03	
		28–34	0.05	0.03	0.03	
9.	MCHC [g/dL]	0–6	33.89	1.39	34.15	0.7422
		7–13	33.58	0.68	33.50	
		14–20	34.10	2.12	34.10	
		21–27	32.15	0.64	32.15	
		28–34	32.93	1.15	32.90	
10.	Total bilirubin [mg/dL]	0–6	0.47	0.17	0.44	0.5041
		7–13	0.51	0.23	0.50	
		14–20	0.18	0.02	0.18	
		21–27	0.39	0.08	0.39	
		28–34	0.49	0.31	0.49	
11.	ESR [mm/h]	0–6	22.44	18.95	24.00	0.1483
		7–13	19.00	10.65	15.00	
		14–20	29.50	6.36	29.50	
		21–27	18.50	23.33	18.50	
		28–34	35.00	46.03	12.00	

* Av–average, EO–Eosinophils, ESR–Erythrocyte Sedimentation Rate, IG–Immature Granulocyte Count, MCHC–Mean Corpuscular Haemoglobin Concentration, Me–Median, MONO–Monocytes, NEUT–Neutrophils, NEUT-GI–Neutrophil Granularity Intensity, NEUT-RI–Neutrophil Reactive Intensity, PDAI–Pemphigus Disease Area Index, RE-LYMP–Reactive Lymphocytes, SD–Standard Deviation, WBC–White Blood Cells.

## Data Availability

The data used in this study involved confidential patient records from Independent Public Clinical Hospital No. 1 in Lublin. Therefore, they can only be shared with the hospital’s permission. Please contact the hospital’s data protection officer or the director of medical affairs (ul. Staszica 16, 20-081 Lublin, szpital@spsk1.lublin.pl) for reference.
